# Potential Arrival Pathway for Highly Pathogenic Avian Influenza H5N1 to Oceania

**DOI:** 10.1111/irv.70055

**Published:** 2024-12-16

**Authors:** Pablo Plaza, Andrea Santangeli, Tommaso Cancellario, Sergio Lambertucci

**Affiliations:** ^1^ Grupo de Investigaciones en Biología de la Conservación, Laboratorio Ecotono, INIBIOMA Universidad Nacional del Comahue‐CONICET San Carlos de Bariloche Argentina; ^2^ Animal Demography and Ecology Unit Institute for Mediterranean Studies (IMEDEA) Esporles Spain; ^3^ FitzPatrick Institute of African Ornithology University of Cape Town Cape Town South Africa; ^4^ Balearic Biodiversity Centre, Department of Biology University of the Balearic Islands Palma Spain

**Keywords:** H5N1, Maps, Oceania, Risk

In late 2020, the Highly Pathogenic Avian Influenza A(H5N1) (hereafter, H5N1) fired the most severe panzootic ever recorded, causing alarming mortalities in wildlife and domestic animals, with an increasing risk to humans [1, 2, 3, 4]. Almost the entire world has been affected by H5N1; the virus has expanded to new regions such as the Americas and Antarctica for the first time in its evolutionary history [3]. However, no cases of H5N1 have been detected in Oceania to date [5, 6] (only one human case infected outside this continent has been reported [7]). Regions not affected by this virus are of epidemiological importance, as they provide insights about potential limiting factors for its spread (e.g., geographic barriers, environmental features, wild species traits and movement). Moreover, in those areas, there is still time to prepare efficient preventive and mitigation actions to reduce the impact of this pathogen, if we can identify potential pathways of virus arrival. Here, leveraging range maps of suitable host bird species, we suggest a potential pathway of H5N1 arrival to the Oceania region that could be important to consider under the current epidemiological behavior of this virus.

To assess possible pathways of H5N1 arrival to Oceania (specifically, Australia, New Zealand, and Tasmania for this article), we performed a map of risk based on wild bird species already reported as infected by the virus anywhere in the world. These species could be considered suitable hosts of the virus. We integrated a list of H5N1‐infected wild bird species reported in the World Animal Health Information System (WAHIS) database up to April 2024 [3] and Scientific Committee on Antarctic Research up to November 2024 (SCAR) [8], with species distributions primarily based on habitat maps (AOH) [9] and, when these were lacking, bird ranges provided by BirdLife International [10]. We removed records in which infected individuals were not identified at the species level and cases where individuals were kept in captivity. We obtained 345 unique wild bird species found infected by H5N1. To map the risk of H5N1 infection (i.e., areas where species reported as infected are distributed), we used the Additive Benefit Function (ABF) in Zonation v.5 [11].

Our risk map shows that Oceania presents a low risk compared with other regions, because it still does not host many species already reported as infected in the rest of the world (Figure 1A). However, more than 50 species that live in Oceania have already been infected in other regions (Table S1). Many of those species overlap their distributions in most of the coast of Australia and New Zealand, making this region of high risk (Figure 1B). Some key susceptible species reported infected in other regions (e.g., Antarctica and sub‐Antarctic islands) such as Brown skuas ( *Stercorarius antarcticus* ), South Polar skuas ( *Stercorarius maccormicki* ), Wandering albatross ( *Diomedea exulans* ), and Giant petrels ( *Macronectes giganteus* ) are present in the south of Oceania (Figure 1A). These species, and especially immature birds, have large movement patterns (thousands of kilometers), covering areas all around the world at high latitudes [12] (Figure 1A). For instance, immature Wandering Albatrosses tagged in their first year could perform circumnavigations of the globe and travel up to 185,000 km [12, 13] (Figure 1A); individuals from this species can travel 8.5 million kilometers in their entire life of 50 years [13]. Because species mentioned are susceptible and could transport the virus to distant areas, the risk of its arrival in Oceania throughout the Southern Ocean Flyway in the short to medium term is rapidly increasing. Previous studies have sampled thousands of individuals of different wild bird species across Australia to evaluate the potential arrival of H5N1 and have proposed the East Australasian flyway as a potential pathway of virus arrival; to date, there is no evidence of any infection with H5N1 in birds there [5, 6]. Although the East Australasian flyway and its bird species could be considered of high risk for virus arrival [5], our map also suggests that other southern flyways routes and the species using them should be considered to predict the potential arrival and introgression of the virus into this continent (Table S1).

**FIGURE 1:**
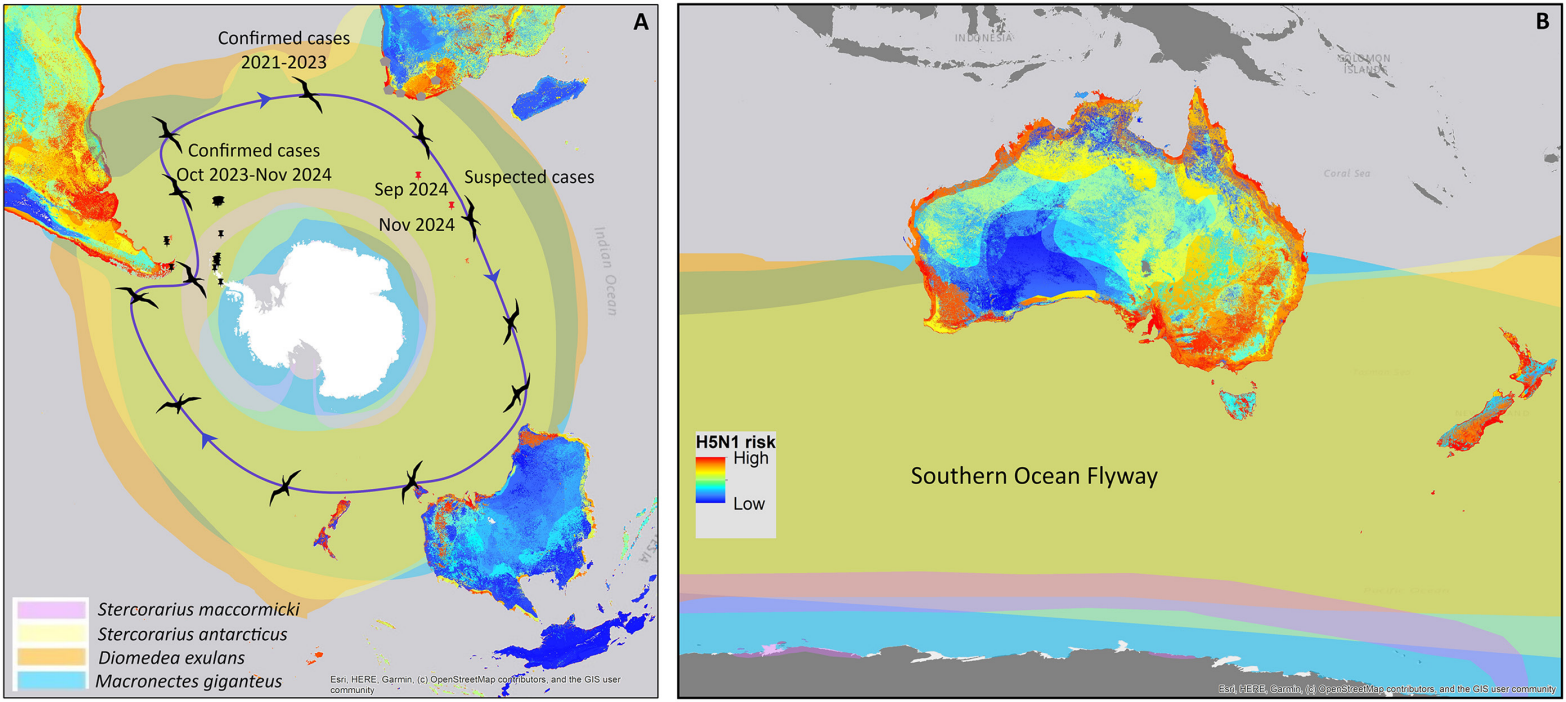
Global risk maps of H5N1 infection based on the distribution range of wild bird species reported as infected between 2020 and 2024 in all continents except Oceania (where the virus is still absent): high risk (red) and low risk (blue). (A) Map of risk including the distribution range of Brown skuas ( *Stercorarius antarcticus* ), South Polar skuas ( *Stercorarius maccormicki* ), Wandering albatross ( *Diomedea exulans* ), and Giant petrels ( *Macronectes giganteus* ), species that already tested positive to H5N1 and move all throughout the Southern Ocean Flyway. Black points indicate suspected and confirmed H5N1 cases in various wild bird species from October 2023 to November 2024, dark grey points positive cases between 2021 and 2023 in Southern Africa, while red points represent new suspected cases (based on mortalities and symptoms) on Marion Island and Île de la Possession detected between September and November 2024. Bird silhouettes and the blue line represent approximate movement patterns of Wandering Albatrosses based on Weimerskirch et al. [13, 15]. (B) Map of H5N1 risk in Oceania based on the species infected in other regions of the world. Coastal areas, particularly in the southern region, are at higher risk due to the presence of species reported as infected in other parts of the world, particularly the species using the Southern Ocean Flyway.

The distribution and movement patterns of Brown, South Polar skuas, Wandering albatross, and Giant petrels encompass Patagonia, in the southern tip of South America, sub‐Antarctic islands, Antarctica, and the southern regions of Oceania [12] (Figure 1A). In Antarctica and the sub‐Antarctic islands, H5N1 cases and suspected infections in these and other wild birds have been reported from 2023 to November 2024; at least 70 cases (confirmed and suspected) were reported in this region [3, 8] (Figure 1A). Worryingly, infected and suspected individuals have been found in new areas to the east of the Antarctic Peninsula, reaching even regions near the south of Africa (Marion Island, −46.876620, 37.744890: three suspected cases, and Ile de la Possession, −46.427645, 51.748694, two suspected cases) between September and November 2024 (Figure 1A) [8]. The virus has potentially traveled around 5000 km in less than 1 year, moving from Bird Island (−54.006869, −38.036471) in October 2023 to the suspected cases of Marion Island in September 2024, primarily associated with skuas, but also with other marine birds [8] (Figure 1A). Although the distance from Marion Island to Australia is approximately 6500 km, the species mentioned above have wide movement patterns (Figure 1A); thus, those distances may only represent a temporary barrier. This was the case of South America, where the virus traveled approximately 8000 km from the Pacific to the Atlantic Ocean, devastating pinnipeds (e.g., *Otaria flavescens* ) populations along its trajectory in less than 1 year [14]. In fact, the nearest distance between the coast of Antarctica to Australia and New Zealand is approximately only 3000 and 2600 km, respectively; thus, if infected birds spread across the Antarctic continent to the east, Oceania could also be at high risk via this pathway.

The epidemiological behavior of the currently circulating H5N1 lineage is continuously changing; the potential for its arrival in Oceania via the Southern Ocean Flyway could be possible, as shown in Figure 1A,B. Therefore, Oceania, the last continent free of this highly dangerous pathogen, is at potential risk of arrival through migratory birds using the Pacific Ocean (East Asian Australasian Flyway) and via the Southern Ocean Flyway (Figure 1B). Our maps show that susceptible host species are present all around the continent but particularly in the south (Figure 1B). Considering they are connected with individuals from other regions [13, 15], they can be infected in some of the places they overlap and act as the pathway for H5N1 to reach this region.

The information provided here could be useful for authorities from the countries in Oceania to focus on implementing surveillance programs taking into account the species and geographical areas of risk suggested here. It is crucial to be well prepared in advance with all the information on potential infection pathways to have better possibilities to deal with this highly virulent and contagious pathogen. Once arrived, this virus can decimate large populations of wild birds and mammals, production systems including poultry and dairy farms, and may even cause human infections [16, 17]. A transboundary coordinated effort is fundamental to deal with H5N1 spread; our main effort should be to limit the arrival of H5N1 to new geographical areas as much as possible at the same time of preparing the regions to reduce the spread as soon as it arrives. To this end, knowing the potential sites, species that are potential vectors of the virus and their ecological behavior, would be an advantage toward containing and mitigating this emerging pathogen that is causing devastating economic and environmental effects globally.

## Author Contributions

**Pablo Plaza:** conceptualization, data curation, investigation, project administration, resources, validation, visualization, roles/writing–original draft, writing–review and editing. **Andrea Santangeli:** conceptualization, data curation, investigation, project administration, resources, validation, visualization, roles/writing–original draft, writing–review and editing. **Tommaso Cancellario:** data curation, resources, validation, visualization, writing–review and editing. **Sergio Lambertucci**: conceptualization, data curation, funding acquisition, investigation, project administration, resources, supervision, validation, visualization, roles/writing–original draft, writing–review and editing.

## Ethics Statement

The authors have nothing to report.

## Consent

The authors have nothing to report.

## Conflicts of Interest

The authors declare no conflicts of interest.

### Peer Review

The peer review history for this article is available at https://www.webofscience.com/api/gateway/wos/peer‐review/10.1111/irv.70055.

## Supporting information


**Table S1** List of wild bird species reported as infected by WAHIS and SCAR from October 2020 to November 2024 in other parts of the world, but also present in Oceania without reports of H5N1 infection.

## Data Availability

The authors have nothing to report.
