# Supplementary material for: Potential Arrival Pathway for Highly Pathogenic Avian Influenza H5N1 to Oceania
Source: Influenza Other Respir Viruses. 2024 Dec 16;18(12):e70055. doi: 10.1111/irv.70055 (PMC11649835; doi:10.1111/irv.70055)
Supplement: Supplementary file 1 — Table S1 List of wild bird species reported as infected by WAHIS and SCAR from October 2020 to November 2024 in other parts of the world, but also present in Oceania without reports of H5N1 infection. [file IRV-18-e70055-s001.docx]

**Table S1:** List of wild bird species reported as infected by WAHIS and SCAR from October 2020 to November 2024 in other parts of the world, but also present in Oceania without reports of H5N1 infection.

| **Scientific name** | **Common name** | **Family** |
| --- | --- | --- |
| *Ardea alba* | Great White Egret | Ardeidae |
| *Ardenna grisea* | Sooty Shearwater | Procellariidae |
| *Ardenna tenuirostris* | Short-tailed Shearwater | Procellariidae |
| *Arenaria interpres* | Ruddy Turnstone | Scolopacidae |
| *Bubulcus ibis* | Cattle Egret | Ardeidae |
| *Calidris alba* | Sanderling | Scolopacidae |
| *Calidris alpina* | Dunlin | Scolopacidae |
| *Calidris canutus* | Red Knot | Scolopacidae |
| *Chlidonias hybrida* | Whiskered Tern | Laridae |
| *Circus assimilis* | Spotted Harrier | Accipitridae |
| *Cygnus atratus* | Black Swan | Anatidae |
| *Diomedea exulans* | Wandering albatross | Diomedeidae |
| *Egretta garzetta* | Little Egret | Ardeidae |
| *Eudyptes chrysocome* | Southern Rockhopper Penguin | Spheniscidae |
| *Falco peregrinus* | Peregrine Falcon | Falconidae |
| *Fregata minor* | Great Frigatebird | Fregatidae |
| *Fulica atra* | Eurasian Coot | Rallidae |
| *Fulmarus glacialoides* | Southern Fulmar | Procellariidae |
| *Gelochelidon nilotica* | Common Gull-billed Tern | Laridae |
| *Hydroprogne caspia* | Caspian Tern | Laridae |
| *Larus dominicanus* | Kelp Gull | Laridae |
| *Larus novaehollandiae* | Silver Gull | Laridae |
| *Leucocarbo atriceps* | Imperial Shag | Phalacrocoracidae |
| *Leucophaeus atricilla* | Laughing Gull | Laridae |
| *Macronectes giganteus* | Southern Giant Petrel | Procellariidae |
| *Macronectes halli* | Northern Giant Petrel | Procellariidae |
| *Milvus migrans* | Black Kite | Accipitridae |
| *Numenius phaeopus* | Whimbrel | Scolopacidae |
| *Pachyptila desolata* | Antarctic Prion | Procellariidae |
| *Pandion haliaetus* | Osprey | Pandionidae |
| *Phalacrocorax carbo* | Great Cormorant | Phalacrocoracidae |
| *Phalacrocorax punctatus* | Spotted Shag | Phalacrocoracidae |
| *Plegadis falcinellus* | Glossy Ibis | Threskiornithidae |
| *Pluvialis squatarola* | Grey Plover | Charadriidae |
| *Podiceps cristatus* | Great Crested Grebe | Podicipedidae |
| *Procellaria aequinoctialis* | White-chinned Petrel | Procellariidae |
| *Pterodroma macroptera* | Great-winged Petrel | Procellariidae |
| *Spatula rhynchotis* | Australasian Shoveler | Anatidae |
| *Stercorarius antarcticus* | Brown Skua | Stercorariidae |
| *Stercorarius maccormicki* | South Polar Skua | Stercorariidae |
| *Stercorarius parasiticus* | Arctic Jaeger | Stercorariidae |
| *Sterna dougallii* | Roseate Tern | Laridae |
| *Sterna hirundo* | Common Tern | Laridae |
| *Sterna paradisaea* | Arctic Tern | Laridae |
| *Sternula albifrons* | Little Tern | Laridae |
| *Streptopelia decaocto* | Eurasian Collared-dove | Columbidae |
| *Sula leucogaster* | Brown Booby | Sulidae |
| *Sula sula* | Red-footed Booby | Sulidae |
| *Thalassarche melanophris* | Black-browed Albatross | Diomedeidae |
| *Thalasseus bergii* | Greater Crested Tern | Laridae |
| *Tyto alba* | Barn-owl | Tytonidae |
